# Study on the Mechanism of *Paeoniflorin*, an Active Component of *Paeonia lactiflora Pall.*, in Improving Skin Pigmentation by Inhibiting the TNF-α Signaling Pathway

**DOI:** 10.3390/ph19030443

**Published:** 2026-03-09

**Authors:** Kela Yin, Song Wang, Weina Wang, Tingting Liu, Dejun Qi, Wei Wang, Marwan M. A. Rashed, Hong Duan, Chenghui He, Mengxiao Zhang, Hao Liu, Kefeng Zhai

**Affiliations:** 1 School of Pharmacy, Bengbu Medical University, Bengbu 233030, China; 18583317378@163.com (K.Y.); 15555302981@163.com (S.W.); zhangmx@bbmc.edu.cn (M.Z.); 2 School of Biological and Food Engineering, Engineering Research Center for Development and High Value Utilization of Genuine Medicinal Materials in North Anhui Province, Suzhou University, Suzhou 234000, China; 17682770813@163.com (W.W.); 17205694920@163.com (T.L.); 13966304864@163.com (D.Q.); marwanrashed6@ahszu.edu.cn (M.M.A.R.); szxydh@163.com (H.D.); 3 Nutrition and Bromatology Group, Department of Analytical Chemistry and Food Science, Faculty of Food Science and Technology, University of Vigo-Ourense Campus, E-32004 Ourense, Spain; 4 Key Laboratory of Xinjiang Uygur Medicine, Xinjiang Institute of Materia Medica, Urumqi 830010, China; hchxjywyjs@163.com

**Keywords:** *Paeoniflorin*, melanin production, TNF-α signaling pathway, network pharmacology, molecular docking, regulation

## Abstract

**Background/Objectives**: This article employs both in vivo and in vitro experiments. **Methods**: The core targets and key pathways of *Paeoniflorin* were predicted using a PPI network analysis, GO analysis, and KEGG analysis. Subsequently, molecular docking analysis and molecular simulation dynamics were performed on the core effector. In vitro experiments employed a UVB-irradiated B16F10 cell model. The effects of Paeoniflorin on melanin content and tyrosinase activity were evaluated. Apoptosis and inflammatory cytokine levels were also assessed in vitro. In vivo experiments used a model combining progesterone injection with UV irradiation. Histopathological skin changes and melanin granule distribution were examined using HE staining. Skin melanin content, tyrosinase activity, and expression levels of related proteins were measured. Additionally, ELISA assays measured serum IL-6 and TNF-α inflammatory cytokines in mice. **Results**: Rese screening identified 69 targets involved in Paeoniflorin’s effects on melanogenesis, including TNF-α, IL-6, TP53, MAPK3, HIF1A and BCL2. Molecular docking and molecular dynamics simulations indicate that Paeoniflorin exhibits strong affinity for tumor necrosis factor-α. In in vitro experiments, Paeoniflorin significantly reduced UVB-induced melanin content and tyrosinase activity in B16F10 cells. It also promoted melanocyte apoptosis and a dose-dependent decrease in IL-6 and TNF-α levels. In vivo, Paeoniflorin significantly reduced epidermal and dermal thickness and inhibited inflammatory infiltration. It decreased melanin granules, melanin content, tyrosinase activity, and IL-6 and TNF-α levels in mouse skin tissue. **Conclusions**: This research indicates that Paeoniflorin can significantly suppress UVB-induced cellular inflammatory responses by inhibiting the TNF signaling pathway, thereby reducing hyperpigmentation.

## 1. Introduction

Skin color is determined by the types and accumulation levels of melanin [1]. Melanin not only dictates skin tone, but also protects against harmful ultraviolet radiation through its accumulation [2]. However, excessive melanin accumulation can lead to various skin issues, such as freckles and melasma, as well as psychological concerns [3]. In recent years, extensive research on melanin has been reported. Professor Gertrude-E Costin’s research indicates that the pigmentary effect of blue light is mediated through calcium-/calmodulin-dependent protein kinase II and MAPK signaling pathways, as well as clusterin-dependent autophagy inhibition mediated by OPN3-TRPV1-calcium influx [4]. Professor Yang Cheng’s team proposed a novel theory based on the inherent cell-to-cell communication mechanism between keratinocytes and melanocytes in the skin’s basal layer. This theory employs biomimetic cell membrane intervention to regulate the transport of melanosomes between keratinocytes and melanocytes, thereby achieving safer and more effective skin-whitening strategies [5]. Professor Hu Yunxia’s research indicates that fibroblasts and hair follicle bulge cells act through a synergistic mechanism involving two signaling pathways: COL6A3-CD44 and SEMA3C-NRP1. These pathways regulate melanocyte survival, migration, and the formation of hair pigment patterns [6]. Both active ingredients, VY-8 and FF-6, are derived from turmeric. They inhibit pigmentation by competitively inhibiting tyrosinase activity and melanin protein synthesis [7]. Professor Wang Guangli’s research indicates that, under low-temperature conditions, PnWRKY19-3 in purple bamboo epidermis promotes tyrosine synthesis by upregulating T5H gene expression, thereby regulating melanin accumulation in the epidermis [8]. The aforementioned research systematically elucidates the multifaceted regulatory mechanisms of melanin synthesis under non-inflammatory conditions from perspectives including physical stimuli, intercellular communication, natural active ingredients, and environmental factors, providing theoretical support for the development of skin-lightening strategies. However, in both physiological and pathological skin processes, inflammatory stress represents another core trigger for pigment metabolic disorders. Post-inflammatory hyperpigmentation (PIH), resulting from abnormal melanin synthesis mediated by inflammation, constitutes a clinically prevalent and challenging-to-treat hyperpigmentation disorder [9]. Research into its specific regulatory pathways and intervention targets remains to be further explored.

In recent years, as research into melanogenesis has deepened, the role of inflammation has attracted increasing attention. Inflammation not only stimulates melanin synthesis, but also produces reactive oxygen species that indirectly promote pigmentation. *Paeonia lactiflora Pall.* (PL) is one of China’s renowned traditional flowers, belonging to the genus Paeonia within the Ranunculaceae family. Revered as the “King of Flowers” for its large, vibrant blooms, it serves both medicinal and culinary purposes as a quintessential dual-use plant. Its flowers, rhizomes, and petals, when properly processed, offer nutritional and health benefits [10,11]. The Materia Medica with Annotations also records that peony root can “eliminate blood stasis and disperse stagnant blood,” indirectly supporting its efficacy in alleviating skin pigmentation issues caused by blood stasis and inflammatory reactions. In traditional skincare practices, peony flowers are commonly processed into extracts or decoctions for topical application or oral consumption to improve sallow complexions and pigmentation issues. Its whitening properties may be linked to its potential role in regulating skin inflammation [12,13]. Modern pharmacological research confirms that its active components—including Paeoniflorin (PF), Paeoniflorin lactone, and benzyl Paeoniflorin—exhibit multiple pharmacological activities such as anti-inflammatory analgesia, immune modulation, and hepatoprotective and nephroprotective effects. These properties provide a crucial material foundation for natural drug development [14,15,16]. Peonies possess dual medicinal and edible properties. The active compounds found in their flowers can aid in the development of skin-brightening products, expanding their application [17,18]. PF, the primary active component of PL, constitutes over 40% of its content. Research indicates that PF exhibits broad anti-inflammatory and immunomodulatory effects [19]. Recent studies suggest that PF possesses anti-pigmentation properties. However, the precise mechanism by which it inhibits melanogenesis remains incompletely elucidated.

Complex diseases often involve the synergistic disruption of multiple genes and pathways, revealing the limitations of traditional single-target drug development approaches. Network pharmacology, grounded in systems biology principles, integrates multi-omics data to construct interaction networks among drugs, targets, and diseases. This holistic approach unravels the drug’s mechanisms of action, thereby overcoming traditional research bottlenecks [20]. Molecular docking is a core technology in computer-aided drug design. In particular, it simulates binding patterns and affinities between small molecules and biological macromolecular targets. This approach enables the efficient screening of potential active compounds [21]. The integration of network pharmacology and molecular docking forms a “systematic prediction-precise validation” research framework, widely applied in drug development and mechanism exploration [22]. This approach provides robust technical support for our study, aiding in the discovery of potential therapeutic targets and drug action patterns for disease treatment [23].

This study employs a comprehensive approach integrating network pharmacology, molecular docking, and in vitro and in vivo experiments. The aim was to elucidate the molecular mechanism by which PF inhibits melanin deposition. We further examined mechanisms involving the TNF, IL-6, and apoptosis signaling pathways. This analysis clarifies how PF regulates melanin synthesis. This provides an experimental foundation for the addition of whitening chemicals to cosmetics.

## 2. Results

### 2.1. Employing Network Pharmacology to Analyze the Relationship Between PF and Melanin Synthesis

**Figure 1:**
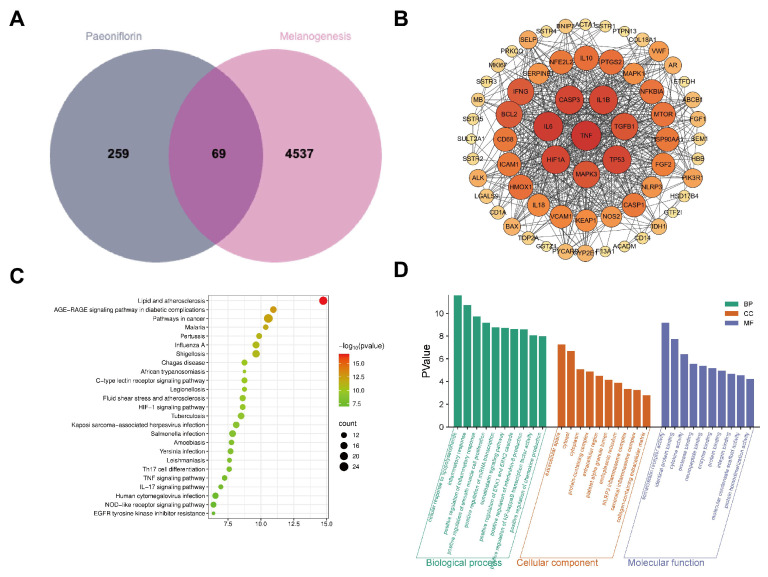
Employing network pharmacology to analyses the relationship between PF and melanin synthesis. (**A**) Venn diagram of Paeoniflorin with melanogenesis-related targets; (**B**) protein–protein interaction network map of Paeoniflorin and melanogenesis-related targets; (**C**) bubble plot of enrichment analyses for Paeoniflorin targeting melanogenesis-related pathways; (**D**) GO enrichment analysis of Paeoniflorin-targeted melanogenesis-related targets.

To investigate potential target sites for PF in inhibiting melanin synthesis, a network pharmacology analysis was conducted. First, using ‘PF’ as the keyword across the CTD, PharmMapper, and TCMSP databases, 328 target sites were identified after integration and deduplication. Concurrently, searching the Genecards database for ‘melanogenesis’ yielded 4606 melanin-related disease target sites. A Venn diagram was constructed using the MicroBioinformatics platform. The results indicate (Figure 1A) that 69 targets are shared between the monomer compounds and the disease. To elucidate the interaction mechanisms between drugs and diseases at the molecular network level of protein interactions, we constructed a protein interaction network diagram of PF and melanogenesis using Cytoscape software. The results show (Figure 1B) that the potential primary targets associated with PF and melanin synthesis included TNF, IL-6, TP53, MAPK3, and HIF1A (Table 1).

To investigate the functional characteristics of differentially expressed genes, we performed GO analysis on 69 intersecting genes associated with PF and melanogenesis using the DAVID database. The results reveal 133 biological process (BP) entries, 43 cellular component (CC) entries, and 42 molecular function (MF) entries (sorted by *p*-value). We selected the top 10 entries for visualization, revealing the following (Figure 1C): among BP entries, major differentially expressed genes were significantly enriched in immunity, inflammatory response, and signal regulation; and in CC entries, major differentially expressed genes focused on intracellular structures and inflammatory complexes, particularly inflammasome assembly and collagen-rich extracellular matrix. MF entries primarily involved signal recognition and molecular interactions, with enriched functions including somatostatin receptor activity, cytokine/neuropeptide binding, and protease activity. KEGG pathway analysis predicted the signaling pathways involved in the intervention. The results reveal 133 pathways (Figure 1D). We ranked the pathways by *p*-value and selected the top 25, among which the TNF signaling pathway and IL-6 signaling pathway were significantly enriched.

### 2.2. Molecular Docking Prediction of PF Inhibiting Melanin Deposition

**Figure 2:**
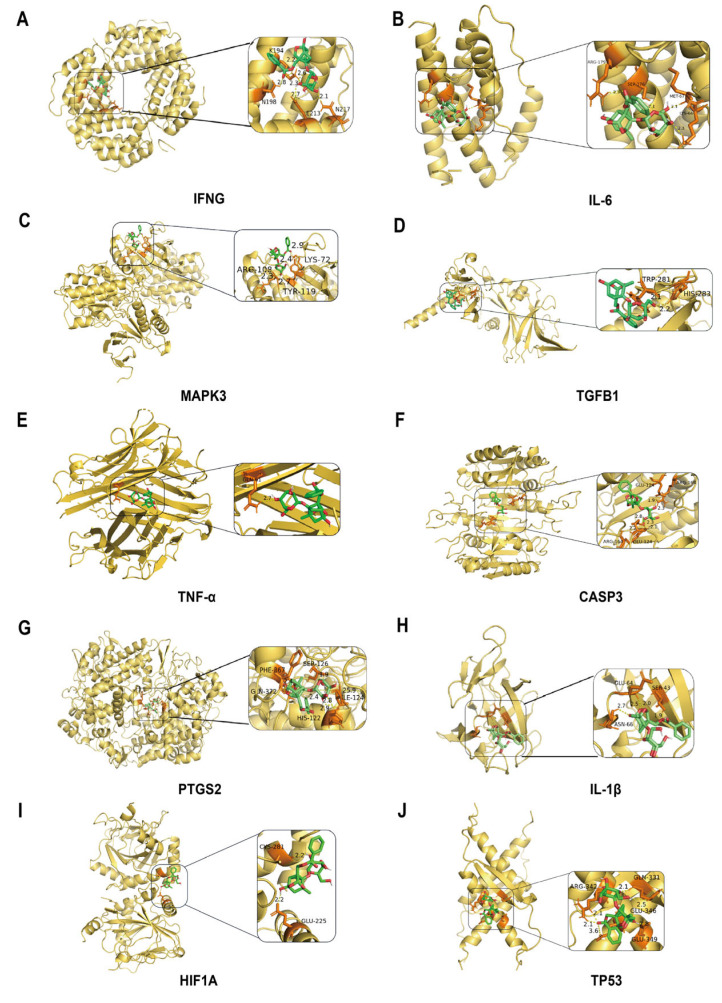
Molecular docking prediction of Paeoniflorin inhibiting melanin deposition. (**A**–**J**) Molecular docking visualization of active ingredients with various target proteins (yellow: proteins; green: small molecules; orange: binding sites).

To further validate the core targets predicted by network pharmacology, we employed molecular docking to predict binding energies between PF and various targets, including IL-1β, IFNG, HIF-1A, CASP3, BCL2, IL-6, TNF-α, TGFB1, PTGS2, and MAPK3. Our results indicate (Figure 2A–J) that the average binding energy between the small molecule and the target protein is −7.35 kcal/mol. Notably, TNF exhibits the strongest interaction with a binding energy of −9.672 kcal/mol, establishing PF as a key ligand for inhibiting melanin synthesis (Table 2).

### 2.3. Molecular Simulation Dynamics Results

**Figure 3:**
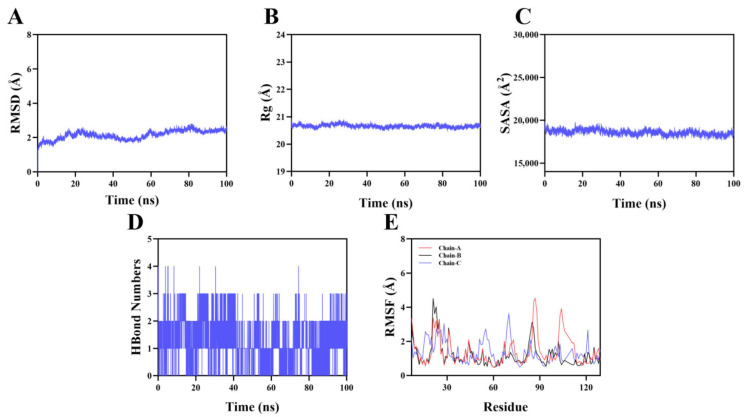
Molecular dynamics simulation of protein–ligand complexes. (**A**) RMSD values of protein–ligand complexes over time; (**B**) Rg values of protein–ligand complexes over time; (**C**) SASA values of protein–ligand complexes over time; (**D**) HBonds values of protein–ligand complexes over time; (**E**) root mean square fluctuation (RMSF) values of protein–ligand complexes.

To validate the stability of the binding mode between Paeoniflorin (PF) and its core target TNF-α (PDB ID: 6OP0), a 100 ns molecular dynamics simulation was performed on the ligand–target complex formed between the two. The results of the molecular dynamics simulation confirmed the stability of the complex binding. As shown in Figure 3A, the RMSD curve indicates that the complex reached kinetic equilibrium at approximately 60 ns, and the value remained stable at around 2.2 Å during the equilibrium period, indicating that the overall conformation was highly stable; in Figure 3B, the Rg value remains constant at 20.5 Å throughout and showed no significant fluctuations, indicating that the binding of the small molecule did not change the overall folding state of the target protein, and the backbone structure was compact; in Figure 3C, the SASA curve fluctuates slightly, further confirming that the microenvironment of the binding interface and the protein surface conformation were in a stable state. The analysis of hydrogen bond numbers (Figure 3D) shows that the number of hydrogen bonds in the complex dynamically maintained between 0 and 4, averaging approximately 2 and persisting continuously, forming a stable hydrogen bond network; the RMSF analysis in Figure 3E further indicates that the flexibility of the vast majority of residues was lower than 3 Å, and the local flexibility was extremely low. In conclusion, the multi-dimensional indicators all confirmed that the conformation of this small molecule and the target protein was stable.

### 2.4. The Melanogenesis-Inhibiting Effect of PF on B16F10 Cells

**Figure 4:**
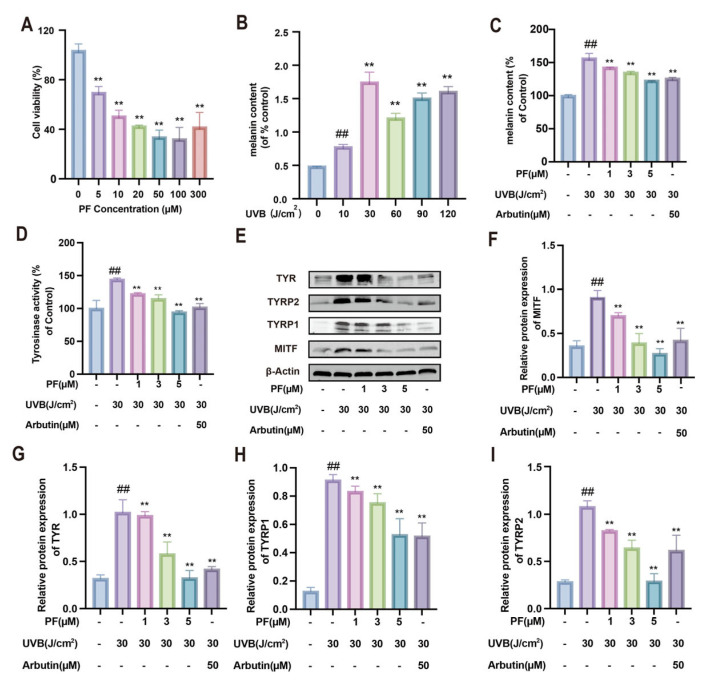
The melanogenesis-inhibiting effect of Paeoniflorin on B16F10 cells. (**A**) Cell viability in groups treated with different concentrations of PF (0–300 μM). (**B**) Melanin content in groups exposed to different doses of UVB (0–120 J/cm^2^). (**C**,**D**) Melanin content and tyrosinase activity in different treatment groups. (**E**–**I**) Detection of proteins related to melanin production (TYR, TYRP2, TYRP1, MITF) and β-actin levels by Western blotting; Analysis of protein expression. Compared to the blank group, ## *p* < 0.01; compared to the UVB-only treatment group, ** *p* < 0.01. ($\bar{x}$ ± s, *n* = 6).

The results indicate (Figure 4A) that significant cytotoxicity was observed at a concentration of 10 μM after B16F10 cells were exposed to PF for 24 h (*p* < 0.01). At concentrations ranging from 5 to 10 μM, cytotoxicity was relatively low, with an IC_50_ of 9.92 μM for cell viability inhibition. Based on these findings, subsequent experiments employed low (1 μM), medium (3 μM), and high (5 μM) doses of PF.

UVB irradiation was used to establish a melanin production model to identify an optimal irradiation dose. The results demonstrate (Figure 4B) that UVB stimulation progressively increased melanin synthesis in B16F10 cells. At an irradiation dose of 30 J/cm^2^, melanin synthesis exhibited significant enhancement (*p* < 0.01). Consequently, subsequent experiments utilized a 30 J/cm^2^ irradiation dose.

Melanin content and tyrosinase activity are key indicators of melanin synthesis. The results reveal that UVB (30 J/cm^2^) stimulation significantly elevated melanin content and tyrosinase activity in the model group compared to the control group (*p* < 0.05). Notably, PF intervention significantly suppressed melanin content and tyrosinase activity (*p* < 0.01), exhibiting a dose-dependent decreasing trend (Figure 4C,D).

To evaluate the regulatory effect of PF on melanin at the protein level, this study examined its impact on melanin signaling-related proteins in B16F10 cells (Figure 4E–I). The results show that, compared to the blank group, the model group exhibited significantly elevated protein levels of MITF, TYR, TYRP1, and TYRP2, indicating that UVB irradiation promotes melanin synthesis in melanocytes. Notably, PF intervention significantly reduced the expression levels of these proteins in the model group (*p* < 0.05). These findings indicate that UV stimulation is a key factor in promoting melanin synthesis by melanocytes. PF can reduce melanin content, tyrosinase activity, and the expression levels of melanin-related proteins. This demonstrates that PF exerts a significant inhibitory effect on melanin at the cellular level.

### 2.5. The Effect of PF on Inhibiting the Regulation of Melanin Synthesis by Targeting TNF and IL-6

**Figure 5:**
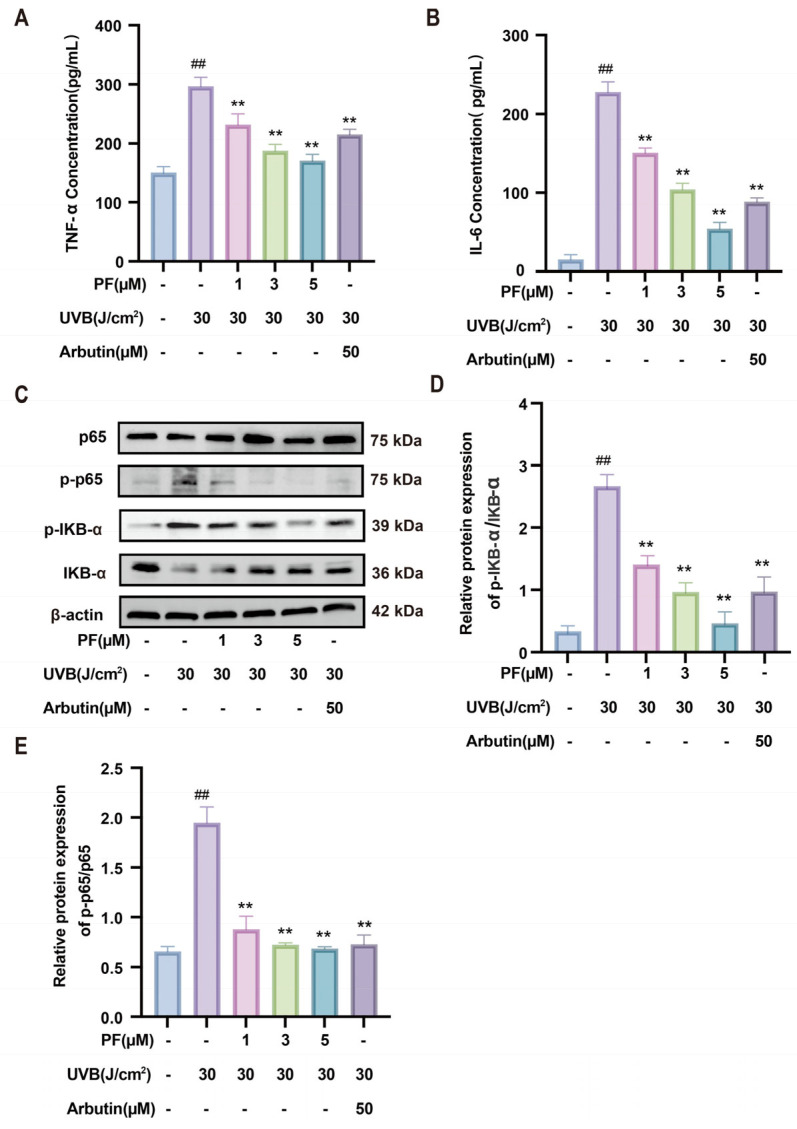
The effect of Paeoniflorin on inhibiting the regulation of melanin synthesis by targeting TNF and IL-6. Detection of inflammatory cytokine concentrations in different treatment groups: (**A**) IL-6 concentration; (**B**) TNF-α concentration. (**C**) Western blot was used to detect the levels of TNF-α signaling pathway-related proteins (p65, p-p65, p-IκB-α, IκB-α) and β-actin. (**D**,**E**) Analysis of relative protein expression levels. Compared to the blank group, ## *p* < 0.01; compared to the UVB-only treatment group, ** *p* < 0.01. ($\bar{x}$ ± s, *n* = 6).

Inflammatory factors directly reflect changes in the level of inflammatory response. It is important to note that inflammatory factors can either directly act on melanocytes or indirectly regulate melanin formation by affecting keratinocytes. As shown in the figure, compared to the blank group, B16F10 cells exposed to 30 J/cm^2^ UVB irradiation exhibited significantly elevated levels of IL-6 and TNF-α. This indicates increased release of inflammatory factors and heightened inflammatory activity during melanin synthesis. Compared to the model group, PF exhibited a dose-dependent inhibition of IL-6 and TNF-α, thereby suppressing inflammatory response levels (Figure 5A,B). This suggests that PF can inhibit inflammatory reactions during melanogenesis, consequently suppressing melanin production.

Further analysis revealed that, compared to the blank group, the model group exhibited significantly elevated expression of, p-IκBα/IκBα, and p-p65/p65 (*p* < 0.05). Following PF intervention, the expression levels of these proteins were significantly reduced (*p* < 0.05) (Figure 5C–E). In summary, PF inhibits melanin production by downregulating the, p-IκBα/IκBα, and p-p65/p65 signaling pathways.

### 2.6. The Effect of PF on UVB-Induced Apoptosis in B16F10 Cells

**Figure 6:**
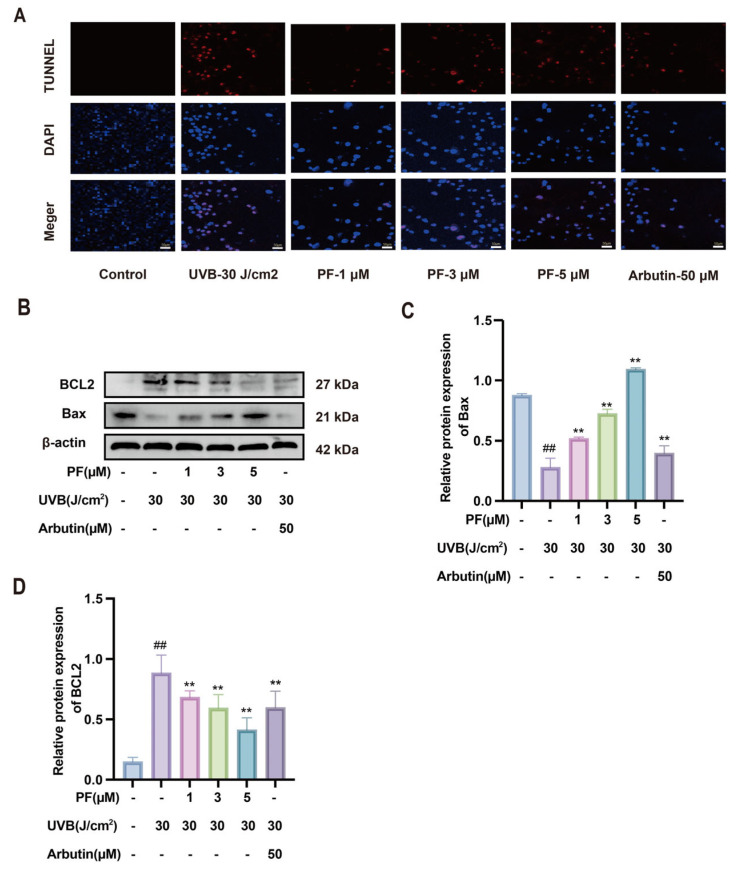
The effect of Paeoniflorin on UVB-induced apoptosis in B16F10 cells. (**A**) TUNEL fluorescence staining was used to detect cell apoptosis in different treatment groups. (**B**) Western blot was used to detect the levels of apoptosis-related proteins (BCL2, Bax) and β-actin. (**C**,**D**) Analysis of relative protein expression levels of Bax (**C**) and BCL2 (**D**). Compared to the blank group, ## *p* < 0.01; compared to the UVB-only treatment group, ** *p* < 0.01. ($\bar{x}$ ± s, *n* = 6).

Inflammation and apoptosis are closely interrelated and mutually regulate key nodes within the cellular stress response network. In the UVB-induced B16F10 process, apoptosis was promoted (Figure 6A). The results indicate that, compared to the blank group, B16F10 cells exposed to 30 J/cm^2^ UVB radiation exhibited significantly reduced apoptosis. In contrast, PF demonstrated a concentration-dependent promotion of apoptosis compared to the model group.

Protein expression levels of the Bax and BCL2 signaling pathways were assessed via Western blot analysis (Figure 6B–D). The results reveal that, compared to the blank group, the model group showed decreased BCL-2 protein expression and increased Bax protein expression. Following PF intervention, the expression levels of these proteins in the model group were significantly reversed. These findings indicate that PF downregulates the anti-apoptotic protein Bax and upregulates the pro-apoptotic protein BCL2, thereby promoting melanocyte apoptosis and inhibiting melanin production.

### 2.7. The Effect of PF on Melanin Accumulation in the Dorsum of Mice

**Figure 7:**
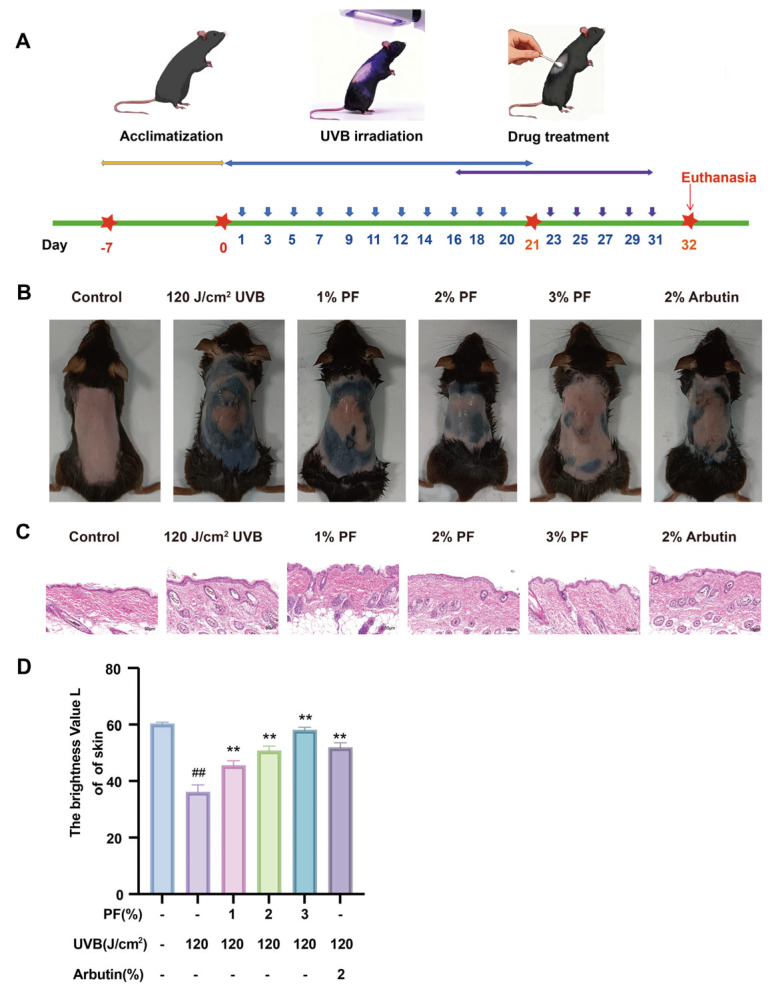
The effect of Paeoniflorin on melanin accumulation in the dorsum of mice (**A**) Schematic Diagram of Mouse Experiment Workflow. (**B**) Melanin production in the back skin of mice. (**C**) HE staining of mouse skin. (**D**) Brightness detection of mouse skin. Compared to the blank (Control) group, ## *p* < 0.01; compared to the UVB-only treatment group, ** *p* < 0.01. ($\bar{x}$ ± s, *n* = 6).

In the animal skin melanin model, experiments typically employ UVB irradiation combined with progesterone injection (Figure 7A). The results show that the control group mice exhibited pale red skin without observable epidermal thickening or pigmentation. In contrast, the model group mice displayed pigmentation, significant epidermal thickening, and mild erythema, indicating successful establishment of the skin melanin model (*p* < 0.01). Following PF intervention, pigment deposition and erythema were significantly improved (*p* < 0.01) (Figure 7B,C). Skin brightness assessment revealed markedly reduced brightness in the model group, which was significantly reversed after PF treatment (Figure 7D).

### 2.8. Effects of PF on Melanin and Tyrosinase Activity in Skin Tissue

**Figure 8:**
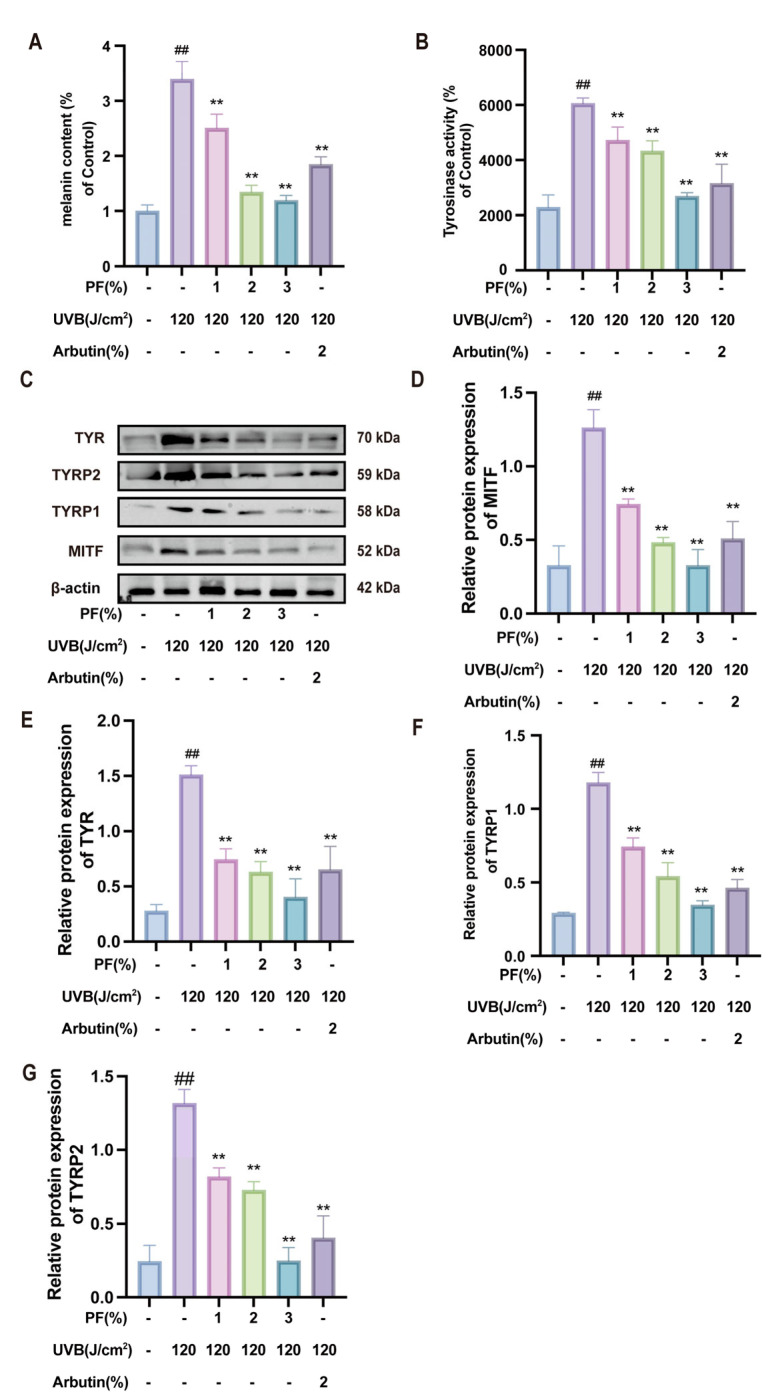
Effects of Paeoniflorin on melanin and tyrosinase activity in skin tissue. (**A**) Quantitative analysis of melanin content in each treatment group. (**B**) Detection of tyrosinase activity in animal skin of different treatment groups. (**C**) Western blot was used to detect the levels of melanogenesis-related proteins (TYR, TYRP2, TYRP1, MITF) and the internal β-actin. (**D**–**G**) Analysis of relative protein expression levels. Compared to the blank (Control) group, ## *p* < 0.01; compared to the UVB-only treatment group, ** *p* < 0.01. ($\bar{x}$ ± s, *n* = 6).

Melanin content and tyrosinase activity in mouse skin serve as key indicators of melanin synthesis. Further results demonstrate that combined UVB irradiation and progesterone injection significantly increased melanin content and tyrosinase activity in the model group (*p* < 0.05). Notably, PF exhibited a marked reversal effect on elevated melanin content and tyrosinase activity (*p* < 0.01), demonstrating a dose-dependent decreasing trend (Figure 8A,B).

Western blot analysis revealed significantly elevated protein expression of MITF, TYR, TYRP1, and TYRP2 in the model group compared to the control group (*p* < 0.01). Following PF intervention, the expression levels of these proteins were significantly reduced (*p* < 0.01) (Figure 8C–G). The results indicate that PF effectively reverses melanin deposition in mouse skin, enhances skin brightness, protects the epidermis, and inhibits melanin protein expression, thereby suppressing melanin production.

### 2.9. Effects of PF on Skin Inflammatory Factors in Mice

**Figure 9:**
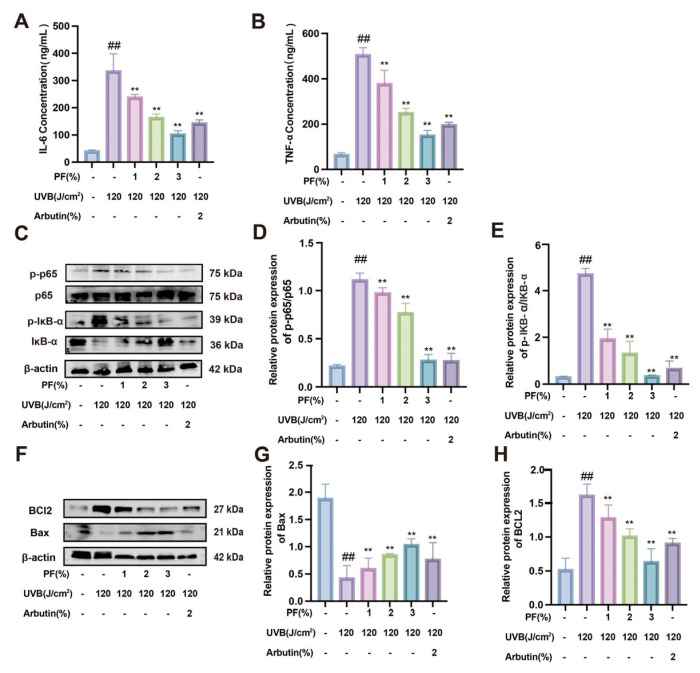
Paeoniflorin alleviates skin inflammation in UV-irradiated mice and modulates apoptosis. (**A**,**B**) Inflammatory cytokine concentration detection in different mouse treatment groups. (**C**) Expression levels of TNF and IL-6 signaling pathway-related proteins (p65, p-p65, p-IκB-α, IκB-α) and β-actin were detected by Western blotting. (**D**,**E**) Analysis of relative protein expression levels: (**F**) Western blot analysis of apoptosis-related proteins (BCL2, Bax) and β-actin expression levels in different treatment groups. (**G**,**H**) Analysis of relative protein expression levels. Compared to the blank group, ## *p* < 0.01; compared to the UVB-only irradiation group, ** *p* < 0.01. (Mean ± SD, *n* = 6).

Following UVB irradiation, significant localized hyperpigmentation developed. This resulted from inflammatory mediators released during the inflammatory process, which directly or indirectly stimulated melanocyte production. The study reveals that serum IL-6 and TNF-α levels in the model group mice were significantly elevated compared to the control group (*p* < 0.01). This indicates that UVB irradiation stimulates the release of inflammatory mediators, thereby triggering an inflammatory response. However, after PF intervention, the levels of inflammatory cytokines in the mice decreased significantly (*p* < 0.01) (Figure 9A,B).

To further validate the mechanism of PF in treating hyperpigmentation, Western blotting was used to detect the expression levels of proteins related to the TNF-α and IL-6 pathways (Figure 9C–E). The results show that, compared to the control group, the model group exhibited significantly elevated expression of p-IκBα/IκBα and p-p65/p65 (*p* < 0.05). Following PF intervention, the expression levels of these inflammation-related proteins were significantly reduced (*p* < 0.05). In summary, ultraviolet irradiation induces an inflammatory response in skin tissue, which subsequently affects epidermal melanin deposition. PF effectively suppresses this inflammatory response by inhibiting the release of inflammatory mediators.

### 2.10. Effects of PF on BCL2 and Bax Protein Levels in UVB-Induced B16F10 Cells

Western blot analysis of Bax (pro-apoptotic) and BCL2 (anti-apoptotic) protein expression levels revealed (Figure 9F–H) that, compared to the blank group, the model group exhibited significantly elevated BCL2 protein expression (*p* < 0.01) and markedly reduced Bax protein expression (*p* < 0.01). Following PF intervention, the expression levels of BCl-2 and Bax proteins showed a significant reversal. This indicates that PF effectively reduces the expression of the anti-apoptotic protein BCL2 while simultaneously increasing the expression of the pro-apoptotic protein Bax. This mechanism promotes melanocyte apoptosis, reduces melanin accumulation on the skin surface, and thereby inhibits melanin production.

## 3. Discussion

Melanin deposition refers to abnormal increases in melanin synthesis by melanocytes and an uneven distribution of melanin due to changes in internal and external environments, leading to abnormal pigment accumulation in the skin [24]. Abnormal melanin deposition can cause skin darkening, hypopigmentation, melasma, and other skin issues [25], and is one of the factors affecting skin esthetics [26]. Research indicates that multiple factors, including genetic predisposition, inflammatory factors, oxidative stress, and tyrosinase-catalyzed reactions, influence the pathogenesis of melanin deposition. These factors can lead to abnormal melanocyte synthesis and trigger various skin diseases [27]. Historically, treatment options for hyperpigmentation were limited. Beyond physical methods such as laser therapy and photorejuvenation, adjunctive pharmacological approaches included vitamin C, corticosteroids, and calcineurin inhibitors [28]. However, traditional treatments often entail high costs and a tendency for post-treatment hyperpigmentation. Paeonia lactiflora is a perennial herbaceous plant in the Fabaceae family, prized for its large, vibrant flowers [29]. PF, a monoterpene glycoside compound and one of the primary constituents of Paeonia lactiflora, serves as the main bioactive component of this plant [30]. PF has been demonstrated to possess multiple pharmacological activities [31], including anti-inflammatory [32], antioxidant [33], immunomodulatory [34], and antitumor effects [35]. Recent studies indicate that PF exerts regulatory effects on skin pigmentation [36,37]. Although its skin-lightening efficacy has been confirmed, the mechanisms underlying its regulation of melanin production remain to be investigated. Therefore, this study aims to explore the mechanism by which PF inhibits melanin deposition.

This study investigates the efficacy and mechanism of PF in inhibiting melanogenesis using network pharmacology and molecular docking combined with in vitro and in vivo experiments. Network pharmacology and molecular docking analyses revealed that PF interacts with 69 melanin-deposition-related targets. Among these, TNF, IL-6, IFNG, IL-1β, TGFB1, PTGS2, BCL2, MAPK3, CASP3, and HIF-1A were selected as core targets for molecular docking. The results indicate higher target relevance for TNF and IL-6. Subsequently, melanin-inhibition effects were evaluated using melanin content, tyrosinase activity, and Western blotting assays.

Melanin deposition is closely associated with inflammatory responses. Inflammation serves as the core factor sustaining melanin metabolic imbalance, and is also the key driver of continuous melanin accumulation [38,39]. TNF and IL-6, pivotal inflammatory mediators in melanin-related inflammation, promote melanin synthesis in melanocytes by releasing inflammatory factors [40], thereby inducing epidermal hyperpigmentation [41,42,43]. When skin is exposed to external environmental stimuli, inflammatory factors are released, stimulating melanin deposition at the skin surface. In our study, B16F10 cells were irradiated at an energy density of 30 J/cm^2^ to establish a melanin model. The results demonstrate significantly elevated levels of TNF and IL-6 in the cells. However, following PF intervention, both melanin content and levels of inflammatory factors in the cells were significantly reduced. This indicates that PF effectively suppresses the release of inflammatory factors, thereby diminishing melanin production.

## 4. Materials and Methods

### 4.1. Cell and Animal Husbandry

B16F10 mouse melanoma cells were purchased from Wuhan Punoise Life Technology Co., Ltd. (Wuhan, China). The cells were cultured in RPMI 1640 medium supplemented with 10% fetal bovine serum and 1% antibiotic-antifungal solution. Thirty-six SPF-grade female C57BL/6 mice, aged 8 weeks and weighing 20–23 g, were purchased from Henan Skebes Biotechnology Co., Ltd. (Zhengzhou, China). They were acclimatized at the Animal Experiment Center of Suzhou University under conditions of 20–25 °C and a 12 h light–dark cycle for one week. Animal experiments were conducted in strict compliance with the Guide for the Care and Use of Laboratory Animals published by the National Institutes of Health, and were approved by the Animal Ethics Committee of Suzhou University (Approval No.: ssxyll-2025022).

### 4.2. Medicines and Reagents

PF (Plant Origin Bioligical Co., Ltd., Nanjing, China; CAS: 23180-57-6); Fetal Bovine Serum (FBS, NSERA, Catalog No.: S711-001S); Arbutin (Nanjing Yfxbio Biotech Co., Ltd., Nanjing, China; Catalog No.: 497-76-7); BCA Protein Concentration Assay Kit (Cat. No.: E112-01); Mouse Tumor Necrosis Factor Alpha (TNF-α) ELISA Kit (Nanjing Yfxbio Biotech Co., Ltd., Nanjing, China; Cat. No.: YFXEM00031); Mouse Interleukin-6 (IL-6) ELISA Kit (Nanjing Yfxbio Biotech Co., Ltd., Nanjing, China; Catalog No.: YFXEM00045); TUNEL Apoptosis Assay Kit (UElandy; Shanghai, China; Catalog No.: T6014L); RIPA Lysis Buffer (Nanjing Novazene Biotechnology Co., Ltd., Nanjing, China; Catalog No.: E311-02); Primary antibodies included MITF (Abmart, Shanghai, China; Catalog No.: TP53061S); TYRP1 (Abmart, Shanghai, China; Catalog No.: PC15510S); TYR (Proteintech, Wuhan, China; Catalog No.: 31291-1-AP); TYRP2 (Abways, Shanghai, China; Catalog No.: AY1921); Bax (Proteintech, Wuhan, China; Catalog No.: 50599-2-Ig); BCL2 (Bioworld; Beijing, China; Catalog No.: BS40178); IκB-α (Bioworld; Beijing, China; Catalog No.: AP0634); p-IκB-α (Bioworld; Beijing, China; Catalog No.: BS4105); NF-κB-p65 (Bioworld; Beijing, China; Catalog No.: BS1253); p-NF-κB-p65 (Bioworld; Beijing, China; Catalog No.: BS4136); β-Actin (Proteintech; Wuhan, China; Catalog No.: 20536-1-AP).

### 4.3. Predicting PF and Melanin Deposition Targets

Using “PF” as a small-molecule search term, predict the action targets of active compounds via the CTD database (https://ctdbase.org/, accessed on 14 June 2025), PharmMapper database (http://lilab-ecust.cn/pharmmapper/, accessed on 14 June 2025), and TCMSP platform (https://www.tcmsp-e.com, accessed on 14 June 2025). Using “melanogenesis” as a disease search term, screen disease targets in the Genecards database (https://www.genecards.org/, accessed on 14 June 2025). Finally, compile information on PF and melanogenesis targets and generate a Venn diagram using the online tool Draw Venn Diagram (https://www.bioinformatics.com.cn/, accessed on 14 June 2025).

### 4.4. Protein–Protein Interaction (PPI) Network Construction

To reveal functional collaborative relationships among intracellular proteins at the holistic level and aid in understanding the interactions between drugs and disease targets, the intersecting target dataset was imported into the STRING database. Species parameters were set to “Homo sapiens,” interaction score thresholds were set to 0.7, and discrete protein targets were hidden to construct the PPI network. Using Cytoscape 3.9.1 software, the intersecting target dataset was imported to construct a “component-disease-target” network. Topological analysis was performed, including degree, betweenness centrality, and closeness centrality, to screen targets.

### 4.5. Gene Ontology (GO) and Kyoto Encyclopedia of Genes and Genomes (KEGG) Pathway Analysis

To elucidate its biological mechanisms, we performed GO and KEGG enrichment analyses on the potential targets of PF. We uploaded the targets to the DAVID database (https://davidbioinformatics.nih.gov/, accessed on 14 June 2025) and set the species to “human.” The GO analysis covered three major domains: molecular function (MF), cellular component (CC), and biological process (BP). KEGG pathway analysis screened the top 20 enriched pathways based on *p* < 0.1 and FDR < 0.05 criteria, followed by visualization via the MicroBioinformatics platform (https://www.bioinformatics.com.cn/, accessed on 14 June 2025).

### 4.6. Molecular Docking

Molecular docking was performed on key targets identified through degree-based ranking and KEGG pathway analysis. The two-dimensional structure of Paeoniflorin was downloaded from the TCMSP database and optimized using Chem3D software 17.0. Protein models were obtained from the PDB database and optimized using Pymol 2.3.0 software (deionization and dehydration). The protein–small molecule complex was saved in pdbqt format using Autodock 1.5.7. Docking grid boxes were constructed by defining atomic types, and results were exported in pdbqt format. The Autodock Vina scoring function was employed to evaluate the affinity of the generated ligand binding sites. All reference ligands were redocked with an RMSD < 1.5 Å. The grid size was uniformly set to 40 × 40 × 40 Å, utilizing Autodock Vina’s default 0.675 Å resolution to balance computational accuracy and efficiency, ensuring coverage of the active site and surrounding flexible regions. PyMOL 2.3.0 was used to visualize the conformation with the lowest binding energy, prioritizing thermodynamic stability. A lower binding energy indicates greater stability in the ligand–receptor interaction.

### 4.7. Molecular Simulation Dynamics

The research system for molecular dynamics (MD) simulation is the ligand–target complex formed by Paeoniflorin (PF) and TNF-α (PDB ID: 6OP0). This system is the core complex selected based on the results of molecular docking (the binding energy between PF and TNF-α is −9.672 kcal/mol, indicating the strongest binding affinity among all targets). It is also the core target of the key signaling pathway (TNF-α signaling pathway) focused on in this study. In this study, molecular dynamics simulations were performed using GROMACS 2022. Ligand topologies were generated with the GAFF2 force field via the sobtop_1.0 (dev3.1) program, and atomic charges were assigned using the RESP method. The receptor protein was modeled with the AMBER14SB force field. The system was solvated in a cubic water box with a 1 nm edge length using the TIP3P water model, and 0.15 M NaCl ions were added with the gmx genion tool 2024.6 to maintain electroneutrality. Long-range electrostatic interactions were treated with the Particle Mesh Ewald (PME) method (cutoff distance: 1 nm), and bond constraints were applied using the LINCS algorithm. Prior to simulation, energy minimization was carried out in three stages: 3000 steps of steepest descent followed by 2000 steps of conjugate gradient. The production simulation was conducted under the NPT ensemble for 100 ns with a 2 fs integration time step. Temperature and pressure were maintained at 310 K (using the Nosé–Hoover thermostat) and 1 bar (using the Parrinello–Rahman barostat), respectively. System properties, including root mean square deviation (RMSD), root mean square fluctuation (RMSF), hydrogen bonds (HBonds), radius of gyration (Rg), and solvent-accessible surface area (SASA), were computed using relevant GROMACS tools (2024.6).

### 4.8. Cell Culture

The B16F10 cell line was purchased from Procell Biotechnology Co., Ltd. (Wuhan, China). All cells were cultured in RPMI1640 medium supplemented with 10% fetal bovine serum (FBS) and 1% penicillin/streptomycin. Cells were maintained at 37 °C with 5% CO_2_.

### 4.9. UVB-Irradiated Cells

When the cell confluence reached approximately 60%, the medium was replaced. Cells were then irradiated with UVB in the dark at 0, 30, 60, 90, and 120 J/cm^2^. Irradiation time was calculated using the following formula:

(1) Dose=Time×Intensity

The UV lamp was positioned at a calibrated distance to ensure constant intensity. After irradiation, the medium was replaced and the cells were cultured for 24 h. The following day, discard the old medium, wash three times with PBS, then proceed with the subsequent experimental steps for each experimental group and calculate melanin content.

### 4.10. CCK8 Assay

B16F10 cells in the logarithmic growth phase were seeded at 5 × 10^3^ cells per well in 96-well plates. Cells were cultured at 37 °C with 5% CO_2_ for 24 h. After attachment, the medium was replaced. Intervention groups received PF at 0, 5, 10, 20, 50, 100, and 300 μmol/mL. After 48 h of incubation, the CCK8 reagent was added at a 10% of the medium volume. Cells were incubated in the dark for 1–4 h. Optical density (OD) was measured at 450 nm using a microplate reader and cell survival rates was then calculated.

### 4.11. Experimental Groups

Cells were randomly assigned to groups and cultured in 1640 medium. Untreated cells served as the blank group, while cells exposed to UVB at 30 J/cm^2^ and cultured in serum-free 1640 medium constituted the model group. Drug treatment groups: After UVB induction at 30 J/cm^2^, solutions containing 1.0, 3.0, and 5.0 μmol/mL PF were added. Positive control group: Cells were cultured in serum-free 1640 medium containing 50.0 μmol/mL arbutin. After 24 h of sterile incubation, the experiments commenced.

### 4.12. Effect of PF on Melanin Content in B16F10 Cells

Intracellular melanin content was determined using the NaOH lysis method. B16F10 cells were seeded at 2 × 10^5^ cells/well in six-well plates. Cells were cultured at 37 °C with 5% CO_2_ for 48 h. Cells were then treated according to the experimental groups, with three replicate per group. After 48 h, add 1 mol/L NaOH solution was gently added. Samples were gently mixed and incubated in an 80 °C water bath for 60 min of lysis. Cells were centrifuged at 12,000 rpm for 10 min; the supernatant was then transferred to a 96-well plate. Absorbance was measured at 490 nm, and cellular melanin content was finally calculated.

### 4.13. Effects of PF on Tyrosinase Activity in B16F10 Cells

The intracellular tyrosinase activity was determined by the L-DOPA oxidation method: The cell grouping method was as described above. B16F10 cells were seeded at a density of 2 × 10^5^ cells per well in a six-well plate and cultured at 37 °C with 5% CO_2_ for 48 h. Then, 500 μL of PBS solution containing 1% Triton X-100 was added. The samples were frozen at −80 °C for 2 h, thawed, and lysed at 37 °C for 30 min. Subsequently, 50 μL of L-DOPA (4 mmol/L) was added, and the mixture was incubated at 37 °C for 1 h. Finally, the supernatant was transferred to a 96-well plate, and the absorbance value was measured at 475 nm. Cell tyrosinase activity was calculated using the provided formula.

### 4.14. Effects of PF on UVB-Induced Apoptosis in B16F10 Cells

Cells were grouped according to the aforementioned method, with each group undergoing three replicate experiments. After 48 h of drug treatment, experiments were conducted according to the protocol of the apoptosis assay kit.

### 4.15. Effects of PF on UVB-Induced IL-6 and TNF-α Expression in B16F10 Cells

Cells were grouped as described above, with three replicates per group. Then, 48 h after drug treatment, IL-6 and TNF-α levels were quantified using ELISA kits according to the manufacturer’s protocols.

### 4.16. Western Blot

Following drug treatment, total protein was extracted from cells using a cell lysis buffer, and protein concentration was determined using a BCA protein assay kit. Proteins were separated by SDS-PAGE electrophoresis, transferred to a PVDF membrane, and blocked with 5% bovine serum albumin (BSA) at room temperature for 2 h. The membrane was then washed three times with TBST and incubated overnight with the primary antibody at 4 °C. The next day, membranes were washed and incubated with horseradish peroxidase (HRP)-labeled secondary antibody at room temperature for 2 h. Following this, the membrane was washed three times with TBST. Immunoreactive bands were then visualized using an enhanced chemiluminescence (ECL) detection kit. Images were captured using a gel imaging system, and ImageJ software 1.8.0 was employed to measure the gray values of the target protein relative to the reference protein for normalization, enabling quantitative analysis of band intensity.

### 4.17. Preparation of Sample Solutions

In total, 0.2 g of CMC-Na was added to 5 g of glycerol, then mixed thoroughly. Depending on the group assignment, 20 mL of pure water was added to the blank group and model group. PF administration group: Add 0.2 g, 0.3 g, and 0.4 g of PF, respectively, along with 20 mL of pure water. Positive drug group: Add 0.3 g arbutin and purified water, then continue grinding in one direction until a gel-like consistency is achieved. Remove and bottle the mixture.

### 4.18. Animal Model Establishment

Eight-week-old female C57BL/6J mice were randomly divided into six groups (n = 6 per group): control, model, model + PF (1%, 2%, 3%), and model + arbutin (2%). A 3 × 3 cm area on the dorsal surface was depilated using a commercial depilatory cream. A mouse model of skin hyperpigmentation was established by intramuscular injection of progesterone into the hindlimbs, followed by ultraviolet irradiation. The model + PF groups received daily topical application of PF at designated concentrations for three consecutive weeks.

### 4.19. Mouse Skin Luminance Detection

For each experimental group, 3 cm × 3 cm skin areas were randomly marked on each mouse. The measurement environment was kept dark. Mice were lightly anesthetized with isoflurane before measurement. A calibrated skin color difference meter was placed vertically at the center of each marked area. The measurement button was pressed to record values. Measurements were repeated from three to five times at different locations. All measurement data were recorded.

### 4.20. H&E Staining

Immerse the harvested mouse skin tissue in formalin and dehydrate using a sucrose gradient. Embedded specimens were mounted on a cryostat for sectioning. Sections were fixed in 4% paraformaldehyde for 20 min, washed three times with PBS, stained with hematoxylin and eosin solutions, dehydrated in a graded ethanol series, mounted with neutral resin, and photographed.

### 4.21. Melanin Content Detection

Mouse skin tissue samples were collected and extraction solution was added at a mass-to-volume ratio of 1:5–1:10. Samples were homogenized on ice. Homogenates were centrifuged at 12,000 rpm for 20 min at 4 °C. The supernatant was collected, one molar NaOH was added, and the mixture was gently mixed by pipetting. Samples were incubated at 80 °C for 60 min to achieve cell lysis. The supernatant was transferred to a 96-well plate. Absorbance was measured at 490 nm.

### 4.22. Tyrosinase Activity Assay

Mouse skin tissues were collected, and the extraction solution was added at a mass-to-volume ratio of 1:5–1:10. Samples were homogenized in an ice bath. Homogenates were centrifuged at 12,000 rpm for 20 min at 4 °C. The supernatant was collected and kept on ice. Subsequent steps followed the instructions for the tyrosinase activity assay kit. The kit was purchased from Beijing Solabio Biotechnology Co., Ltd. (Beijing, China) for subsequent steps.

### 4.23. ELISA Assay for IL-6 and TNF-α Inflammatory Cytokines

Skin tissue specimens were weighed after sectioning. A measured volume of PBS was added. Samples were thoroughly homogenized by hand or with a homogenizer. Homogenates were centrifuged at 2000–3000 rpm for approximately 20 min. The supernatant was carefully collected. One portion was used immediately for analysis. The remaining samples were frozen for later use. Subsequent procedures were performed according to the ELISA kit’s instructions.

### 4.24. Cell Apoptosis

Skin tissue was obtained from mice using the aforementioned method. Paraffin sectioning was performed in accordance with the protocol provided by the apoptosis assay kit.

### 4.25. Analysis of Data

All experiments were independently repeated three times and analyzed statistically. Data were processed using GraphPad Prism 9.5.0 software, with statistical significance set at *p* < 0.05. Data are expressed as mean ± standard deviation. Intergroup comparisons were performed using one-way analysis of variance (ANOVA) and Tukey’s post hoc test.

## 5. Conclusions

This study employs network pharmacology and molecular docking analysis to investigate PF’s core targets in melanogenesis, revealing its association with IL-6, TNF, and apoptosis-related signaling pathways. This indicates that PF suppresses hyperpigmentation by regulating the IL-6 and TNF pathways to inhibit inflammatory mediator release, promote apoptosis, and inhibit melanocyte synthesis, thereby achieving a hyperpigmentation-inhibiting effect.

## Figures and Tables

**Table 1:** PF and melanin protein interaction network (top 10 degree values).

NO.	Name	Degree
1	TNF	42
2	IL-6	40
3	IL-1β	40
4	HIF-1A	40
5	CASP3	40
6	TP53	37
7	TGFB1	37
8	MAPK3	36
9	IFNG	36
10	BCL2	32

**Table 2:** Binding energy of Paeoniflorin molecules with core target sites (kcal/mol).

Protein	Bind Energy (kcal/mol)
TNF(PDB ID:6OP0)	−9.672
TGFB1(PDB ID:5VQP)	−7.654
TP53(PDB ID:7DVD)	−8.967
MAPK3(PDB ID:2ZOQ)	−7.786
IFNG(PDB ID:1EKU)	−7.224
IL-6(PDB ID:1ALU)	−7.137
IL-1β(PDB ID:1HIB)	−6.718
HIF-1A(PDB ID:5L9V)	−7.864
CASP3(PDB ID:7RNG)	−8.532
BCL2(PDB ID:1G5M)	−7.893

## Data Availability

The original contributions presented in this study are included in the article. Further inquiries can be directed to the corresponding author.

## Abbreviations

The following abbreviations are used in this manuscript:

PF	Paeoniflori
TNF-α	tumor necrosis factor alpha
IL-6	interleukin-6z
GO	Gene Ontology
KEGG	Kyoto Encyclopedia of Genes and Genomes
BP	biological process
CC	cellular component
MF	molecular function
MIT	melanocyte inducing transcription factor
TYR	Tyrosinase
TYRP1	Tyrosinase-Related Protein 1
TYRP2	Tyrosinase-Related Protein 2
IκBα	Inhibitor of Nuclear Factor κBα
p-IκB -α	Phosphorylated Inhibitor of Nuclear Factor κBα
NF-κB-P65	Nuclear Factor κB p65 Subunit
P- NF-κB-P65	Phosphorylated Nuclear Factor κB p65 Subunit
β-actin	beta-actin
BCL2	B-cell lymphoma 2 protein
Bax	BCL2-associated X protein
CCK-8	Counting Kit-8
PIH	Post-Inflammatory Hyperpigmentation
